# Effective orifice area after Myval and SAPIEN transcatheter valve implantation stratified by flow status: insights from the LANDMARK trial

**DOI:** 10.1016/j.ijcha.2026.101966

**Published:** 2026-06-29

**Authors:** Akihiro Tobe, Yoshinobu Onuma, Niels van Royen, Ignacio J. Amat-Santos, Pieter C. Smits, Marie-Claude Morice, Andreas Baumbach, Patrick W. Serruys

**Affiliations:** a CORRIB Research Centre for Advanced Imaging and Core Lab, University of Galway, Galway, Ireland; b Department of Cardiology, Radboud University Hospital, Nijmegen, the Netherlands; c Centro de Investigación Biomédica en Red - Enfermedades Cardiovasculares (CIBERCV), Instituto de Salud Carlos III, Madrid, Spain; d Department of Cardiology, Hospital Clínico Universitario de Valladolid, Valladolid, Spain; e Cardiovascular European Research Center (CERC), Massy, France; f ICPS, Hôpital privé Jacques Cartier, Massy, France; g Centre for Cardiovascular Medicine and Devices, William Harvey Research Institute, Queen Mary University of London and Barts Heart Centre, London, United Kingdom; h Cleveland Clinic, London, United Kingdom

**Keywords:** Transcatheter aortic valve replacement, Aortic stenosis, Flow status, Effective orifice area, Myval, Sapien

## Correspondence

Transcatheter aortic valve replacement (TAVR) is a guideline-endorsed treatment for severe aortic stenosis [1]. Effective orifice area (EOA) is a key measure of the hemodynamic performance of the transcatheter heart valves (THVs). EOA is assessed by the Doppler echocardiography and is known to be flow dependent. A substudy of the PARTNER trials demonstrated higher EOA in normal- versus low-flow patients across all valve sizes after SAPIEN series implantation [2].

Recently, Krasniqi L et al. reported the postprocedural echocardiographic outcomes of the Myval and SAPIEN series stratified by flow status from the COMPARE-TAVI trial [3]. Given that the Myval THV series is a relatively novel device with still limited echocardiographic evidence, independent validation from additional randomized trials remains important. We therefore evaluated the postprocedural EOA of the balloon-expandable Myval and SAPIEN series from the LANDMARK trial stratified by flow status.

Details of LANDMARK trial are published elsewhere [4], [5]. Briefly, this was a randomized, multicenter, non-inferiority trial comparing Myval THV series with the contemporary THVs (SAPIEN/Evolut series). 768 patients with symptomatic severe native aortic stenosis were randomized 1:1 ratio to Myval or contemporary THV group. The Myval group received Myval or Myval Octacor, whereas the SAPIEN group received SAPIEN 3 or Ultra.

In this substudy, analysis was performed in the as treated population in the Myval and SAPIEN groups. Postprocedural EOA and stroke volume index (SVi) were assessed by echocardiography 1 month after TAVR (or predischarge if unavailable). EOA and SVi were paired at the same echocardiographic examination. Patients without EOA or SVi data were excluded. EOA values for each valve size were stratified according to SVi: low (<35 ml/m^2^), normal (35–50 ml/m^2^), and high (>50 ml/m^2^). Results were presented for overall cohort and for each valve size. One patient who received an extra-large size Myval (30.5 mm) was excluded due to protocol deviation.

EOA was calculated using the continuity equation: EOA = (left ventricular outflow tract [LVOT] area * LVOT velocity-time integral [VTI])/ aortic valve [AV] VTI). The LVOT diameter was measured outer edge–to–outer edge at the ventricular end of the stent in the parasternal long-axis view during mid-systole, and LVOT area was calculated as π*(LVOT diameter/2) [2]. The LVOT VTI was obtained just proximal to the stent frame in the apical five- or three-chamber view using pulse-wave Doppler, and AV VTI was measured across the prosthetic valve using continuous-wave Doppler. The SVi was calculated as (LVOT area * LVOT VTI) / BSA, and Doppler velocity index (DVI) as LVOT VTI / AV VTI. All echocardiograms were analyzed at an independent core lab (CORRIB core lab, Galway, Ireland).

Continuous variables were presented as mean ± standard deviation and compared between two groups using Welch's *t*-test. P for trend across valve sizes was calculated using linear regression analysis with valve size treated as a continuous variable.

Postprocedural EOA, mean pressure gradient (MPG), and DVI stratified by valve size and flow status were analyzed in 355 patients who underwent Myval series implantation (Table 1). One-month echocardiographic data were used in 330 patients, whereas predischarge data were used in 25 patients. EOAs showed a stepwise increase in accordance with increasing valve size, whereas MPG showed a stepwise decrease. DVI values were generally similar across valve size. Patients with normal flow showed larger EOAs than those with low flow, but smaller EOAs than those with high flow. Among patients with normal flow, the postprocedural EOAs for 20 mm (*n* = 1), 21.5 mm (*n* = 12), 23 mm (29), 24.5 mm (*n* = 48), 26 mm (45), 27.5 mm (*n* = 36), and 29 mm (*n* = 13) valves were 1.40, 1.57 ± 0.53, 1.87 ± 0.41, 2.00 ± 0.42, 2.21 ± 0.51, 2.24 ± 0.43, 2.66 ± 0.66 cm^2^, respectively. The results for the SAPIEN group (*n* = 193) are shown in Table 2. One-month data were used in 173 patients, whereas predischarge data were used in 20 patients. In the overall cohort (all flow), the Myval group showed a larger EOA (2.05 ± 0.56 vs 1.81 ± 0.50 cm^2^, *p* < 0.0001), lower MPG (8.16 ± 3.53 vs 10.16 ± 4.84 mmHg, p < 0.0001) and higher DVI (0.55 ± 0.13 vs 0.48 ± 0.12, p < 0.0001) than the SAPIEN group. A consistent pattern was observed across all flow subgroups. In low-flow patients, EOA 1.77 ± 0.53 vs 1.61 ± 0.45 cm^2^ (*p* = 0.0290), MPG 7.38 ± 3.76 vs 8.95 ± 3.91 mmHg (*p* = 0.0071), and higher DVI 0.49 ± 0.14 vs 0.44 ± 0.11 (*p* = 0.0075). In normal-flow patients, EOA 2.09 ± 0.52 vs 1.88 ± 0.47 cm^2^ (*p* = 0.0009), MPG 8.10 ± 3.24 vs 10.25 ± 4.12 mmHg (*p* < 0.0001), and DVI 0.56 ± 0.13 vs 0.50 ± 0.12 (*p* = 0.0003). In high flow patients, EOA 2.37 ± 0.52 vs 2.09 ± 0.53 cm^2^ (*p* = 0.018), MPG 9.61 ± 3.56 vs 12.93 ± 7.22 mmHg (*p* = 0.0204), and DVI 0.62 ± 0.12 vs 0.51 ± 0.11 (p < 0.0001).

**Table 1:** EOA, MPG, and DVI of Myval series stratified by flow status.

Myval series						
EOA						
Size (mm)	All flow	Normal (35–50 ml/m^2^)	Low (<35 ml/m^2^)	P⁎ (normal vs low)	High (>50 ml/m^2^)	P⁎ (normal vs high)
20	1.42 ± 0.05 [4]	1.40 [1]	1.40 ± 0.01 [2]		1.50 [1]	
21.5	1.55 ± 0.49 [17]	1.57 ± 0.53 [12]	1.22 ± 0.17 [3]	0.08	1.94 ± 0.20 [2]	0.14
23	1.82 ± 0.50 [68]	1.87 ± 0.41 [29]	1.49 ± 0.48 [23]	0.004	2.18 ± 0.41 [16]	0.02
24.5	1.99 ± 0.49 [91]	2.00 ± 0.42 [48]	1.71 ± 0.46 [25]	0.01	2.38 ± 0.45 [18]	0.004
26	2.13 ± 0.52 [77]	2.21 ± 0.51 [45]	1.85 ± 0.50 [22]	0.008	2.34 ± 0.40 [10]	0.39
27.5	2.24 ± 0.55 [64]	2.24 ± 0.43 [36]	1.99 ± 0.51 [18]	0.08	2.67 ± 0.78 [10]	0.12
29	2.45 ± 0.61 [34]	2.66 ± 0.66 [13]	2.16 ± 0.56 [13]	0.048	2.57 ± 0.46 [8]	0.71
All sizes	2.05 ± 0.56 [355]	2.09 ± 0.52 [184]	1.77 ± 0.53 [106]	<0.001	2.37 ± 0.52 [65]	<0.001
P for trend#	<0.0001	<0.0001	<0.0001		0.003	
MPG						
Size (mm)	All flow	Normal (35–50 ml/m^2^)	Low (<35 ml/m^2^)	P⁎ (normal vs low)	High (>50 ml/m^2^)	P⁎ (normal vs high)
20	12.47 ± 5.44 [4]	12.90 [1]	8.55 ± 1.63 [2]		19.90 [1]	
21.5	11.38 ± 4.40 [17]	11.59 ± 4.85 [12]	9.63 ± 4.05 [3]	0.52	12.75 ± 1.91 [2]	0.58
23	8.69 ± 3.16 [68]	8.57 ± 3.03 [29]	7.88 ± 3.31 [23]	0.45	10.07 ± 2.88 [16]	0.11
24.5	8.63 ± 3.53 [91]	8.74 ± 3.56 [48]	8.17 ± 3.39 [25]	0.51	8.97 ± 3.74 [18]	0.82
26	7.76 ± 3.33 [77]	7.36 ± 2.55 [45]	7.66 ± 4.56 [22]	0.77	9.77 ± 2.82 [10]	0.03
27.5	7.07 ± 3.10 [64]	7.06 ± 1.96 [36]	6.62 ± 4.43 [18]	0.69	7.91 ± 3.76 [10]	0.50
29	6.69 ± 3.17 [34]	6.53 ± 2.84 [13]	4.84 ± 1.47 [13]	0.07	9.94 ± 3.36 [8]	0.03
All sizes	8.16 ± 3.53 [355]	8.10 ± 3.24 [184]	7.38 ± 3.76 [106]	0.10	9.61 ± 3.56 [65]	0.003
P for trend#	<0.0001	<0.0001	0.007		0.07	
DVI						
Size (mm)	all flow	Normal (35–50 ml/m^2^)	Low (<35 ml/m^2^)	P⁎ (normal vs low)	High (>50 ml/m^2^)	P⁎ (normal vs high)
20	0.55 ± 0.13 [4]	0.42 [1]	0.55 ± 0.13 [2]		0.67 [1]	
21.5	0.52 ± 0.17 [17]	0.52 ± 0.18 [12]	0.41 ± 0.09 [3]	0.17	0.67 ± 0.01 [2]	0.02
23	0.53 ± 0.14 [68]	0.56 ± 0.13 [29]	0.44 ± 0.12 [23]	0.001	0.61 ± 0.09 [16]	0.15
24.5	0.56 ± 0.13 [91]	0.56 ± 0.11 [48]	0.49 ± 0.14 [25]	0.07	0.65 ± 0.11 [18]	0.005
26	0.55 ± 0.13 [77]	0.56 ± 0.13 [45]	0.51 ± 0.14 [22]	0.13	0.60 ± 0.11 [10]	0.32
27.5	0.55 ± 0.13 [64]	0.55 ± 0.10 [36]	0.51 ± 0.15 [18]	0.36	0.65 ± 0.16 [10]	0.09
29	0.59 ± 0.14 [34]	0.66 ± 0.15 [13]	0.54 ± 0.11 [13]	0.03	0.56 ± 0.13 [8]	0.12
All sizes	0.55 ± 0.13 [355]	0.56 ± 0.13 [184]	0.49 ± 0.14 [106]	<0.001	0.62 ± 0.12 [65]	<0.001
P for trend#	0.06	0.06	0.04		0.34	

Values are presented with mean ± SD. [n] indicates the number of patients. EOA = effective orifice area, MPG = mean pressure gradient, DVI=Doppler velocity index.

In this study, patients were included only if both EOA and SVi were available from the same echocardiographic assessment at either one month or predischarge. For example, if the 30-day EOA was available but SVi could not be calculated due to missing body weight, the predischarge EOA and SVi were used instead. Therefore, the values may differ slightly from those reported in the main paper published [3], [4].

⁎ *P* values were calculated by *t*-test.

# P for trend was calculated using linear regression analysis, in which valve size was treated as a continuous variable.

**Table 2:** EOA, MPG, and DVI of SAPIEN series stratified by flow status.

SAPIEN series						
EOA						
Size (mm)	All flow	Normal (35–50 ml/m^2^)	Low (<35 ml/m^2^)	P⁎ (normal vs low)	High (>50 ml/m^2^)	P⁎ (normal vs high)
20	1.38 ± 0.36 [7]	1.35 ± 0.33 [4]	1.43 ± 0.48 [3]	0.82		
23	1.58 ± 0.49 [64]	1.76 ± 0.56 [32]	1.31 ± 0.28 [23]	<0.001	1.67 ± 0.31 [9]	0.53
26	1.90 ± 0.45 [86]	1.98 ± 0.38 [41]	1.69 ± 0.48 [29]	0.01	2.09 ± 0.44 [16]	0.38
29	2.06 ± 0.48 [36]	2.12 ± 0.22 [8]	1.86 ± 0.40 [22]	0.03	2.74 ± 0.32 [6]	0.003
All sizes	1.81 ± 0.50 [193]	1.88 ± 0.47 [85]	1.61 ± 0.45 [77]	<0.001	2.09 ± 0.53 [31]	0.052
P for trend#	<0.0001	0.001	<0.0001		<0.0001	
Mean gradient						
Size (mm)	All flow	Normal (35–50 ml/m^2^)	Low (<35 ml/m^2^)	P⁎ (normal vs low)	High (>50 ml/m^2^)	P⁎ (normal vs high)
20	13.14 ± 5.55 [7]	15.90 ± 4.90 [4]	9.47 ± 4.58 [3]	0.14		
23	11.96 ± 5.43 [64]	11.40 ± 4.69 [32]	10.87 ± 3.33 [23]	0.63	16.73 ± 9.29 [9]	0.13
26	9.68 ± 4.47 [86]	9.23 ± 3.15 [41]	8.65 ± 4.43 [29]	0.55	12.69 ± 6.14 [16]	0.046
29	7.55 ± 2.55 [36]	8.07 ± 1.88 [8]	7.28 ± 2.92 [22]	0.39	7.85 ± 1.98 [6]	0.83
All sizes	10.16 ± 4.84 [193]	10.25 ± 4.12 [85]	8.95 ± 3.91 [77]	0.04	12.93 ± 7.22 [31]	0.06
P for trend#	<0.0001	0.0002	0.004		0.02	
DVI						
Size (mm)	all flow	Normal (35–50 ml/m^2^)	Low (<35 ml/m^2^)	P⁎ (normal vs low)	High (>50 ml/m^2^)	P⁎ (normal vs high)
20	0.44 ± 0.12 [7]	0.41 ± 0.10 [4]	0.48 ± 0.16 [3]	0.54		
23	0.46 ± 0.13 [64]	0.50 ± 0.15 [32]	0.40 ± 0.08 [23]	0.002	0.48 ± 0.08 [9]	0.53
26	0.50 ± 0.12 [86]	0.51 ± 0.10 [41]	0.47 ± 0.13 [29]	0.20	0.50 ± 0.11 [16]	0.72
29	0.47 ± 0.10 [36]	0.46 ± 0.05 [8]	0.44 ± 0.10 [22]	0.42	0.60 ± 0.09 [6]	0.01
All sizes	0.48 ± 0.12 [193]	0.50 ± 0.12 [85]	0.44 ± 0.11 [77]	0.003	0.51 ± 0.11 [31]	0.54
P for trend#	0.33	0.72	0.45		0.04	

Values are presented with mean ± SD. [n] indicates the number of patients. EOA = effective orifice area, MPG = mean pressure gradient, DVI=Doppler velocity index.

In this study, patients were included only if both EOA and SVi were available from the same echocardiographic assessment at either one month or predischarge. For example, if the 30-day EOA was available but SVi could not be calculated due to missing body weight, the predischarge EOA and SVi were used instead. Therefore, the values may differ slightly from those reported in the main paper published [3], [4].

⁎ *P* values were calculated by *t*-test.

# P for trend was calculated using linear regression analysis, in which valve size was treated as a continuous variable.

In this substudy of the LANDMARK trial, EOA, MPG and DVI stratified by flow status after implantation of Myval or SAPIEN valves were evaluated, demonstrating that flow status has a significant impact on these hemodynamic parameters. When comparing the echocardiographic performance of different valve types, flow status should therefore be taken into consideration. The Myval series showed higher EOAs than the SAPIEN series across all flow subgroups. However, the SAPIEN 3 Ultra Resilia (S3UR) was not used in the LANDMARK trial. As S3UR has been reported to have a larger EOA and lower MPG than earlier iterations, validation using newer device generations is warranted [6], [7], [8].

Our findings in the Myval group were consistent with the report from the COMPARE-TAVI trial [3]. The relatively large difference between the two studies was observed for the 20 mm valve size: in our analysis, EOA was 1.40 cm^2^ (*n* = 1) in the normal flow group, whereas it was 1.19 ± 0.17 (*n* = 3) in the normal flow group of their study. This numerical difference is most likely attributable to the very small sample size for this valve size, and validation in a larger cohort is required.

## CRediT authorship contribution statement

**Akihiro Tobe:** Conceptualization, Formal analysis, Investigation, Methodology, Project administration, Writing – original draft. **Yoshinobu Onuma:** Resources, Supervision, Writing – review & editing. **Niels van Royen:** Investigation, Writing – review & editing. **Ignacio J. Amat-Santos:** Investigation, Writing – review & editing. **Pieter C. Smits:** Investigation, Writing – review & editing. **Marie-Claude Morice:** Investigation, Writing – review & editing. **Andreas Baumbach:** Investigation, Writing – review & editing. **Patrick W. Serruys:** Supervision, Writing – review & editing.

## Funding

The LANDMARK trial is funded by Meril Life Sciences, India.

## Declaration of competing interest

The authors declare that they have no known competing financial interests or personal relationships that could have appeared to influence the work reported in this paper.
